# Physiological response strategies of pioneer endemic species *Artemisia wudanica* along an aeolian disturbance gradient in semi-arid active sand dunes

**DOI:** 10.3389/fpls.2026.1981049

**Published:** 2026-09-30

**Authors:** Chi Zhang, Haitao Wang, Yongcui Wang, Yu Qiu, Lei Chu, Jianqiang Qian, Quanlai Zhou, Alamusa, Lian Jia, Lixin Wang

**Affiliations:** 1 CAS Key Laboratory of Forest Ecology and Silviculture, Institute of Applied Ecology, Chinese Academy of Sciences, Shenyang, China; 2 University of Chinese Academy of Sciences, Beijing, China; 3 State Key Laboratory of Efficient Production of Forest Resources, Key Laboratory of Forest Cultivation and Management of National Forestry and Grassland Administration, Research Institute of Forestry, Chinese Academy of Forestry, Beijing, China; 4 Tai’an County Jiuguhe Agricultural Development Co., LTD, Anshan, Liaoning, China; 5 College of Forestry, Henan Agricultural University, Zhengzhou, China; 6 College of Chemistry and Life Science, Anshan Normal University, Anshan, Liaoning, China; 7 Department of Earth and Environmental Sciences, Indiana University Indianapolis, Indianapolis, IN, United States

**Keywords:** antioxidative defense, *Artemisia wudanica*, osmotic adjustment, physiological strategy, sand burial, wind erosion

## Abstract

**Introduction:**

Wind erosion and sand burial are recurrent but physically distinct aeolian disturbances in semi-arid dune systems. We investigated how *Artemisia wudanica* seedlings respond physiologically to matched relative gradients of simulated substrate removal and sand burial under controlled pot conditions.

**Methods:**

Seedlings were subjected to five simulated wind-erosion levels (0, 2, 4, 6, and 8 cm) and five sand-burial levels (0, 1.7, 3.3, 5.0, and 6.7 cm). Chlorophyll, proline, soluble sugar, malondialdehyde (MDA), peroxidase (POD), and superoxide dismutase (SOD) were quantified. Treatment effects were evaluated using two-way ANOVA, PCA of standardized variables, disturbance-specific Spearman correlations, and an exploratory path analysis.

**Results:**

Disturbance type, relative disturbance level, and their interaction affected all six physiological traits, and PCA separated the two-treatment series in multivariate space. Under sand burial, chlorophyll, proline, and soluble sugar showed positive within-treatment covariation after Spearman correlations. Under simulated wind erosion, POD and SOD covaried positively, whereas MDA was not significantly correlated with either antioxidant enzyme; chlorophyll–proline and soluble sugar–MDA associations also remained significant after correction. The exploratory path model had limited overall fit and was used only to summarize statistical associations, not to establish causal physiological mechanisms.

**Discussion:**

Under the controlled conditions of this seedling experiment, the two isolated treatments were associated with different physiological response and covariation patterns. These results provide testable hypotheses about disturbance-specific seedling responses rather than evidence of switchable association, population-level persistence, or restoration performance. Validation is required under field conditions, across additional life stages, and with pulsed and combined erosion–burial events.

## 1 Introduction

Semiarid sandy lands are among the most physically dynamic terrestrial environments. On mobile and semi-mobile dunes, recurrent wind erosion and sand burial continually reorganize surface topography and the near-ground environment, thereby regulating plant establishment, persistence, regeneration, and wind-mediated propagule movement (Ba et al., 2024; Ma et al., 2022; Ooi, 2015; Wang et al., 2015; Zhu et al., 2016, Zhu et al., 2019). Plants colonizing these habitats must therefore cope simultaneously with chronic water and nutrient limitation—conditions under which belowground nutrient-acquisition and resource-use strategies are critical (Lambers et al., 2006, Lambers et al., 2008, Lambers et al., 2015)—and with recurrent physical disturbances, including root exposure, abrasion, burial, and restricted soil aeration (Xie et al., 2022). Understanding how pioneer psammophytes maintain function under such interacting constraints is central to explaining vegetation persistence and improving restoration of active dunes. Recent field-scale studies have further shown that sand-stabilization measures can substantially reduce wind erosion, while approaches integrating biocementation with plant-compatible materials highlight the need to balance surface stabilization with soil moisture conditions and vegetation establishment in sandy environments (Li et al., 2025, Li et al., 2026a, Li et al., 2026b).

Although wind erosion and sand burial are often treated as opposite phases of a single aeolian process, they impose fundamentally different physical constraints. Wind erosion removes supporting substrate, disrupts the root–soil interface, exposes roots, accelerates dehydration, and increases mechanical abrasion. Sand burial, in contrast, adds substrate around basal shoots and shallow roots, modifying light availability, gas diffusion, mechanical load, and the thermal and hydrological conditions of the near-root microenvironment (Cao et al., 2024). Consequently, plant responses may depend not only on disturbance intensity but also on disturbance identity; wind erosion and sand burial may act as qualitatively distinct environmental filters rather than as interchangeable positions along a common severity gradient (Cao et al., 2024; Lee et al., 2023).

At the physiological level, tolerance to wind–sand disturbance is likely to depend on coordinated regulation among three functional dimensions: photosynthetic maintenance, osmotic adjustment, and antioxidant defense (Muhammad et al., 2021; Chen et al., 2023; Gaile et al., 2021). Chlorophyll maintenance supports carbon assimilation and provides energy for growth, repair, and post-disturbance recovery, whereas burial-induced changes in light and aeration can directly modify photosynthetic performance (Muhammad et al., 2021; Chen et al., 2023; Gaile et al., 2021); burial depth also strongly influences seedling emergence and early growth in sandy habitats (Ma et al., 2007). Proline and soluble sugars function as compatible solutes that help maintain cellular water status and metabolic stability under dehydration, burial, and substrate instability (Hosseinifard et al., 2022; Gurrieri et al., 2020). Meanwhile, superoxide dismutase (SOD) and peroxidase (POD) constrain reactive oxygen species accumulation, while malondialdehyde (MDA) indicates membrane lipid peroxidation and cellular injury associated with root exposure, mechanical damage, and drought-like stress (Gaile et al., 2021; Mishra et al., 2023; Zulfiqar and Ashraf, 2023). These dimensions are mechanistically linked: photosynthetic disruption alters carbon availability and soluble-sugar metabolism, osmotic imbalance can intensify oxidative stress, and antioxidant systems protect photosynthetic tissues and membrane integrity (Muhammad et al., 2021; Hosseinifard et al., 2022; Gurrieri et al., 2020; Mishra et al., 2023; Zulfiqar and Ashraf, 2023; Zhang et al., 2022).

Previous studies have already directly compared wind erosion and sand burial in psammophytic plants and have documented contrasting effects on growth, water relations, photosynthesis, and physiological traits (Lee et al., 2023; Qu et al., 2022; Zhao et al., 2023; Liu et al., 2014). For *A. wudanica* specifically, earlier work has examined plant performance under wind erosion, sand burial, and their combined occurrence (Liu et al., 2014). The contribution of the present study is therefore not a first comparison of the two disturbance types. Instead, we focus on how multiple physiological traits representing photosynthetic maintenance, osmotic adjustment, oxidative damage, and antioxidant defense vary and covary across field-informed, proportionally matched disturbance gradients. This gradient-based multivariate perspective can help characterize treatment-associated physiological response structure while avoiding claims that the present experiment establishes previously unknown causal mechanisms.

This distinction is ecologically important because dune ecosystem function and community assembly are shaped by disturbance regime and environmental context (Sabo et al., 2024), and broader disturbance research shows that plant responses depend on disturbance type, history, and position along environmental gradients (Jenkins and Parker, 2000; Bunn et al., 2010). Trait-integration frameworks further indicate that persistence in resource-impoverished environments emerges from context-dependent coordination, trade-offs, and reconfiguration among resource-acquisition and resource-use functions (Lambers et al., 2011; Lambers, 2022; Huo et al., 2022). A direct gradient-based comparison within a single species is therefore needed to separate disturbance identity from intensity and to reveal how physiological functions are reorganized under wind erosion versus sand burial (Ma et al., 2023).

*Artemisia wudanica* is a locally endemic pioneer psammophyte that occurs predominantly on mobile and semi-mobile dunes and is therefore repeatedly exposed to both substrate removal and deposition (Hu and Gao, 2024; Niu et al., 2025). By stabilizing loose sand, reducing sediment mobility, and ameliorating near-ground conditions, this species contributes to vegetation establishment and dune restoration. Its persistence and local dominance in highly dynamic habitats suggest substantial physiological plasticity, potentially involving flexible coordination among photosynthetic maintenance, osmotic regulation, and oxidative-stress defense (Ma et al., 2022). However, less attention has been given to how multiple physiological traits representing photosynthetic maintenance, osmotic adjustment, oxidative damage, and antioxidant defense covary across comparable disturbance gradients within the same species (Liu et al., 2014). These characteristics make *A. wudanica* an appropriate model for testing whether a pioneer dune species deploys a generalized stress response or switches among disturbance-specific physiological strategies.

Here, we exposed *A. wudanica* seedlings to five levels of simulated wind erosion and five levels of sand burial and quantified six traits representing photosynthetic maintenance (chlorophyll), osmotic adjustment (proline and soluble sugar), oxidative damage (MDA), and antioxidant defense (POD and SOD). Building on previous studies that demonstrated differential effects of wind erosion and sand burial, we asked: (i) how individual physiological traits vary across comparable relative disturbance gradients; (ii) whether the two disturbance types are associated with distinct multivariate physiological response structures; and (iii) how relationships among physiological traits differ between the two disturbance regimes. We expected simulated wind erosion to be more strongly associated with oxidative-damage and antioxidant responses, whereas sand burial was expected to show stronger associations with photosynthetic maintenance and osmotic adjustment. By integrating multiple physiological dimensions across field-informed disturbance gradients, this study aims to refine understanding of seedling-level physiological responses to contrasting aeolian disturbances in *A. wudanica*.

## 2 Materials and methods

### 2.1 Experimental material pretreatment

Seeds of *A. wudanica* were used as experimental material. After manual selection, plump, uniformly sized seeds free of pests, diseases, and mechanical damage were chosen. Seeds were surface-sterilized in 5% sodium hypochlorite solution for 10 min, rinsed three times with distilled water, and then soaked in 0.1% gibberellic acid (GA_3_) solution for 12 h based on the germination characteristics of this species. The germination substrate consisted of undisturbed sand collected from a wind-eroded area, sieved through 2 mm, and sterilized at 80 °C for 30 min.

Seed germination was conducted in a constant-temperature incubator at 25 °C with a 16 h light/8 h dark photoperiod. When cotyledons were fully expanded and hypocotyl height reached approximately 3 cm, seedlings were transplanted into plastic pots (15 cm in diameter × 20 cm in height), with five seedlings planted per pot. All seedlings were allowed to recover for 48 h before subsequent treatment application.

### 2.2 Experimental design

Field monitoring of aeolian disturbance was conducted from April to May 2025 in mobile sand dunes in Ongniud Banner, Chifeng City, Inner Mongolia, China. Ten fixed monitoring sites were established. At each site, nine graduated steel erosion pins (1.0 cm in diameter and 130 cm in length) were installed at 1 m spacing, with the sampling array oriented with respect to the prevailing wind direction. The pins were inserted approximately 80 cm into the sand to minimize displacement and subsidence. At the beginning of monitoring, the distance between the reference mark top and the soil surface was recorded as the baseline. Subsequent changes in soil-surface position relative to the baseline were used to determine wind-erosion or sand-burial depth. Measurements were conducted at approximately 15-day intervals, and the nine pin measurements at each site were averaged to obtain one site-level estimate for each monitoring occasion.

During the April–May monitoring period, the mean sand-burial depth was 5.37 cm, whereas the mean wind-erosion depth was 6.15 cm. Based on these field observations, 5.0 cm sand burial and 6.0 cm wind erosion were used as operational reference disturbance levels for the controlled experiment. To establish comparable relative disturbance intensities between the two disturbance types, five treatment levels corresponding approximately to 0, 1/3, 2/3, 1, and 4/3 of their respective reference levels were used. Accordingly, the wind-erosion treatments were set at 0, 2, 4, 6, and 8 cm, whereas the sand-burial treatments were set at 0, 1.7, 3.3, 5.0, and 6.7 cm (**Supplementary Table S1**). Thus, although the absolute intervals differed between the wind-erosion and sand-burial treatments, both gradients followed the same proportional classification scheme relative to their respective field-based reference levels.

The wind-erosion and sand-burial treatments were established as two independent experimental series. Each series included its own independently established 0 cm control group. Thus, the 0 cm wind-erosion control and the 0 cm sand-burial control consisted of different pots and seedlings and were not shared between the two experiments.

The wind-erosion experiment therefore comprised five treatment levels (0, 2, 4, 6, and 8 cm), whereas the sand-burial experiment comprised five independently established treatment levels (0, 1.7, 3.3, 5.0, and 6.7 cm).

Each treatment consisted of five independent pots, with five seedlings grown in each pot. For physiological measurements, three pots were selected from the five pots in each treatment, and samples obtained from different pots were regarded as independent biological replicates. Thus, three independent biological replicates were used for the physiological analyses of each treatment (n = 3). Seedlings within the same pot were regarded as subsamples rather than independent biological replicates because they experienced the same pot-level disturbance treatment and growth environment. This sampling scheme was used to avoid pseudoreplication.

### 2.3 Wind erosion and sand burial simulation

The wind-erosion and sand-burial treatments were imposed over a 28-day experimental period. Wind erosion was simulated by manually removing surface sand, whereas sand burial was simulated by evenly adding sand around the seedling base. Rather than imposing the final treatment depth in a single application, the prescribed final erosion or burial depth for each treatment was divided approximately evenly over the 28-day period. Sand was therefore progressively removed or added until the designated final treatment depth was reached on day 28. The soil-surface position was checked daily using a graduated ruler to ensure that the cumulative erosion or burial depth remained close to the scheduled value for each treatment.

All pots received the same watering regime, with 500 mL of water applied to each pot every two days. Soil moisture was not maintained at a fixed target value; instead, water supply was standardized across treatments by using the same watering volume and frequency. The experiment was conducted under natural light and ambient temperature conditions, without artificial manipulation of light or temperature.

The simulated wind-erosion treatment was designed to represent progressive substrate removal and the associated root exposure. It did not directly simulate wind force or sand-blasting abrasion; therefore, these additional components of natural wind erosion were not experimentally manipulated in the present study (**Figure 1**).

**Figure 1:**
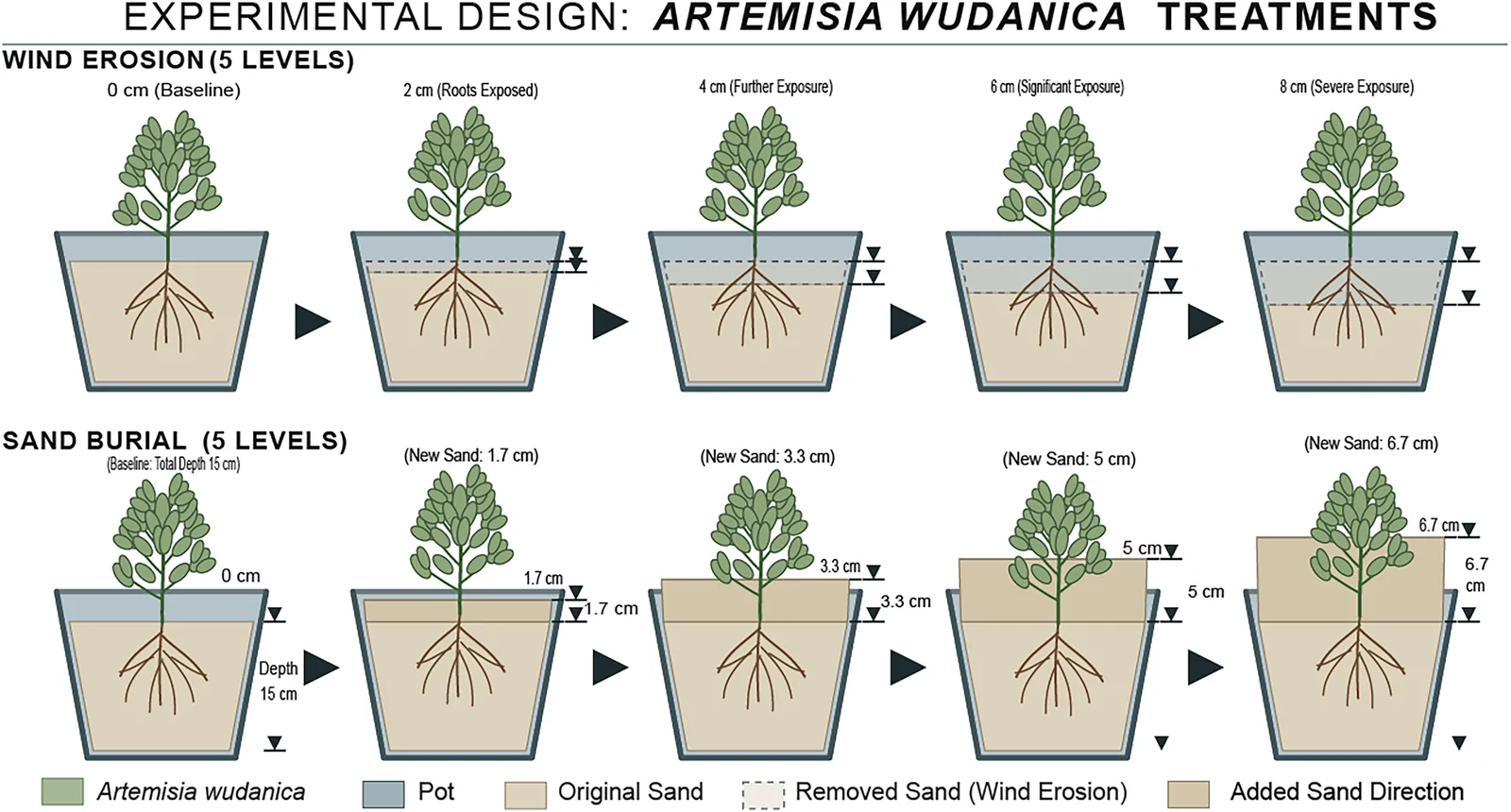
Schematic overview of the experimental system, gradient treatments, and analytical framework used to investigate the physiological responses of *A. wudanica* in response to wind erosion and sand burial.

### 2.4 Measurement of physiological indicators

Six physiological indicators representing three major functional dimensions of plant responses to aeolian disturbance were measured. Chlorophyll content was used as an indicator of photosynthetic maintenance; proline and soluble sugar contents were used to characterize osmotic adjustment; and malondialdehyde (MDA), peroxidase (POD), and superoxide dismutase (SOD) were used to characterize oxidative damage and antioxidant defense.

For physiological measurements, fully mature leaves were randomly collected from the experimental seedlings between 09:00 and 11:00. No fixed leaf position was specified; leaf maturity was used as the primary selection criterion. For each treatment, three independent pots were selected as biological replicates. Within each selected pot, leaves from multiple seedlings were pooled to form one composite sample. Each pot-level composite sample was regarded as one independent biological replicate, resulting in three biological replicates per treatment (n = 3).

Immediately after collection, all leaf samples were flash-frozen in liquid nitrogen. The frozen samples were packed with sufficient dry ice and immediately transported by SF Express cold-chain delivery to Nanjing Hanguang Testing Company (Nanjing, China) for physiological analyses. Transportation was completed within approximately 2 days, and the samples were maintained in a frozen state on dry ice during transportation.

Chlorophyll content, proline content, soluble sugar content, MDA content, POD activity, and SOD activity were subsequently determined by the testing laboratory. Each biological sample was subjected to one analytical determination, with no additional technical replicates. Thus, the three independently sampled pot-level composite samples constituted the three biological replicates used for subsequent statistical analyses. Detailed analytical information for chlorophyll, proline, soluble sugar, MDA, POD, and SOD, including the assay methods, reference standards, instruments, units, and enzyme activity definitions, is provided in **Supplementary Table S2**.

### 2.5 Data analysis

To address our first research question—whether wind erosion and sand burial differ in their effects on each physiological trait of *A. wudanica* across disturbance gradients—all data were first analyzed using two-way analysis of variance (ANOVA), with disturbance type, gradient level, and their interaction as fixed factors. Normality and homogeneity of variance were checked prior to analysis, and Tukey’s HSD test was used for *post-hoc* comparisons (*P* < 0.05). When a significant disturbance type × relative disturbance level interaction was detected, simple-effect analyses were performed using the pooled residual mean square from the full factorial ANOVA. The effect of relative disturbance level was examined separately within each disturbance type, followed by Tukey HSD tests for pairwise comparisons among the five levels (family-wise α = 0.05). To address our second question—whether the two disturbance types generate distinct overall physiological response trajectories—Principal Component Analysis (PCA) was conducted on chlorophyll, proline, soluble sugar, MDA, POD, and SOD. Prior to PCA, each variable was mean-centered and standardized to unit variance using z-score transformation, ensuring that variables measured on different scales contributed comparably to the analysis. PCA scores and loadings were used to visualize multivariate differences among disturbance types and gradients. The PCA was used solely as a descriptive ordination and was not interpreted as evidence of causal or mechanistic relationships. To assess whether physiological traits were coordinated differently under the two disturbance types, Spearman rank correlation analyses were conducted separately for the wind-erosion and sand-burial datasets. For each disturbance type, the analysis included all biological replicates across the five treatment levels (n = 15; five disturbance levels × three biological replicates). Pairwise Spearman correlation coefficients (ρ) were calculated among chlorophyll, proline, soluble sugar, MDA, POD, and SOD. This disturbance-specific analysis was used to avoid correlations caused primarily by between-group differences between wind erosion and sand burial. Finally, an exploratory path analysis was used to examine associations among disturbance type, disturbance gradient, their interaction, and the major physiological response dimensions. Disturbance type was effect-coded and disturbance gradient was mean-centered before constructing the interaction term. Multicollinearity was assessed using variance inflation factors (VIFs), model stability was evaluated using 5,000 bootstrap resamples and leave-one-out sensitivity analysis, and a reduced model was fitted as a parsimony check. Given the relatively small sample size and the limited overall model fit, the path analysis was interpreted as exploratory and was not used to establish causal physiological mechanisms. Together, these analyses enabled us to move beyond single-trait comparisons and evaluate the coordinated strategies by which *A. wudanica* maintains physiological homeostasis under contrasting wind erosion and sand burial conditions.

## 3 Results

### 3.1 Interactive effects of wind erosion and sand burial on physiological indicators

Two-way ANOVA revealed significant effects of disturbance type, relative disturbance level, and their interaction on all six physiological traits (**Supplementary Table S3**). In particular, the disturbance type × relative disturbance level interaction was significant for chlorophyll (F_4,20_ = 15.71, *P* = 5.78 × 10^-6^, ηp^2^ = 0.759), proline (F_4,20_ = 163.49, *P* = 5.67 × 10^-15^, ηp^2^ = 0.970), soluble sugar (F_4,20_ = 189.38, *P* = 1.36 × 10^-15^, ηp^2^ = 0.974), MDA (F_4,20_ = 71.67, *P* = 1.44 × 10^-11^, ηp^2^ = 0.935), POD (F_4,20_ = 366.55, *P* = 2.12 × 10^-18^, ηp^2^ = 0.987), and SOD (F_4,20_ = 273.71, *P* = 3.74 × 10^-17^, ηp^2^ = 0.982). These results indicate that the effect of relative disturbance intensity on each physiological trait depended on disturbance type. Simple-effect analyses further showed significant effects of relative disturbance level within both the wind-erosion and sand-burial treatments for all six physiological traits (**Supplementary Table S4**). Within wind erosion, the simple effects ranged from F_4,20_ = 18.02 for MDA to F_4,20_ = 306.39 for soluble sugar, whereas within sand burial they ranged from F_4,20_ = 54.04 for chlorophyll to F_4,20_ = 455.62 for POD (all *P* < 0.001). Tukey HSD comparisons further identified significant differences among individual relative disturbance levels within each disturbance type (**Supplementary Table S4**).

The significant interactions indicate that the association between relative disturbance level and each measured trait differed between the two treatment series. Among the measured traits, POD and SOD showed the largest interaction effect sizes, indicating particularly strong treatment-specific differences in their response patterns under the conditions tested.

Across gradients, the six traits exhibited contrasting and often non-monotonic response patterns (**Figure 2**). Chlorophyll declined at some lower disturbance levels and increased at higher levels, a pattern consistent with possible compensatory adjustment but not direct evidence of recovery. Proline and soluble sugar tended to be higher under sand burial, whereas MDA, POD, and SOD showed larger fluctuations under simulated wind erosion. These endpoint responses indicate that different physiological traits changed differently across the two isolated treatment series; they do not by themselves establish distinct physiological mechanisms.

**Figure 2:**
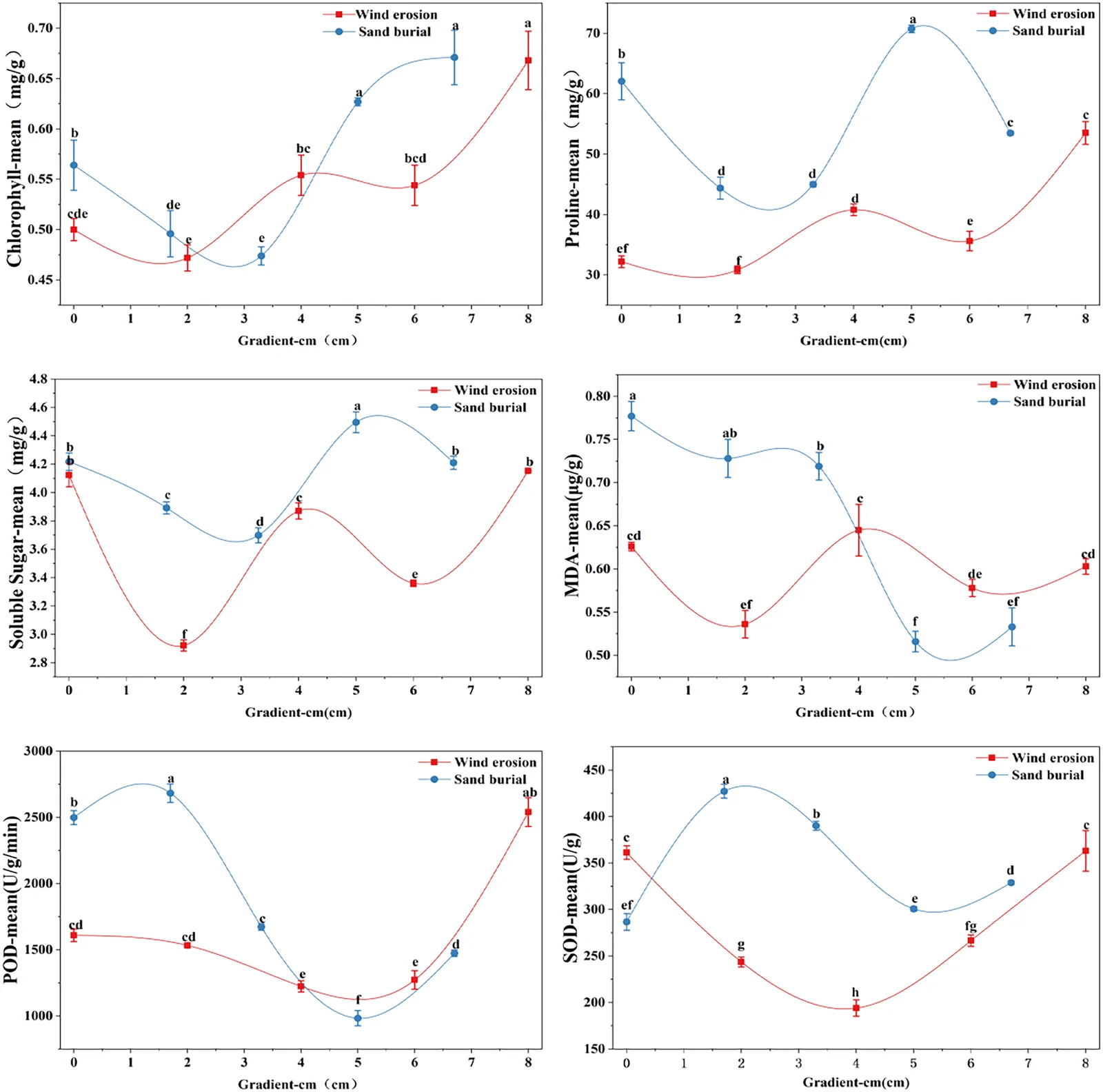
Changes in six physiological indices of *Artemisia wudanica* in response to wind erosion and sand burial gradients. Different lowercase letters indicate significant differences among treatments (two-way ANOVA followed by Tukey’s HSD test, *P* < 0.05). Data are shown as means ± SD (n = 3).

### 3.2 Multivariate response patterns and trait covariation differed between disturbance treatments

The Z-score heatmap showed clear multivariate differentiation between wind erosion and sand burial treatments (**Figure 3**). For descriptive interpretation, chlorophyll represented photosynthetic maintenance, proline and soluble sugar represented osmotic adjustment, MDA represented oxidative damage, and POD and SOD represented antioxidant defense. Sand-burial treatments were more strongly associated with the photosynthetic and osmotic traits, whereas higher simulated wind erosion was more strongly associated with MDA and antioxidant-enzyme responses. These groupings are descriptive functional categories rather than experimentally validated regulatory modules.

**Figure 3:**
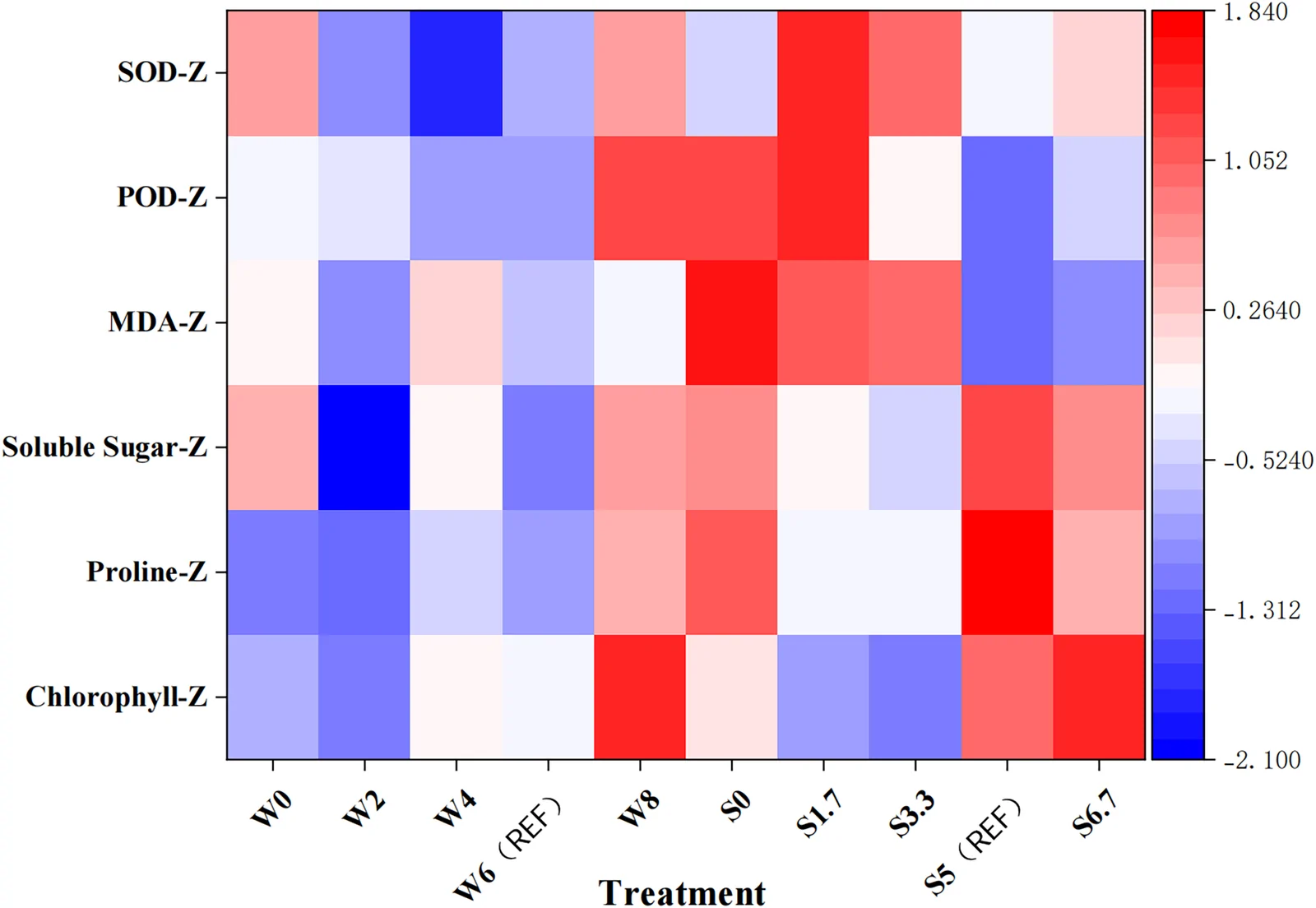
Z-score heatmap of six physiological indicators of *A. wudanica* under wind erosion (W) and sand burial (S) gradients. W0 and S0 are no-disturbance controls; W6 and S5 are the field-based reference treatments (REF).

PCA provided a complementary descriptive view of this divergence (**Figure 4**). PC1 and PC2 explained 40.5% and 35.0% of the total variance, respectively, with a cumulative explained variance of 75.5%. Samples from the two treatment series occupied different regions of ordination space. Chlorophyll, proline, and soluble sugar loaded mainly toward the sand-burial group, whereas MDA, POD, and SOD contributed more strongly to the separation of simulated wind-erosion treatments. Because PCA is an ordination of standardized endpoint measurements, this separation is interpreted as multivariate response differentiation rather than evidence of distinct causal pathways.

**Figure 4:**
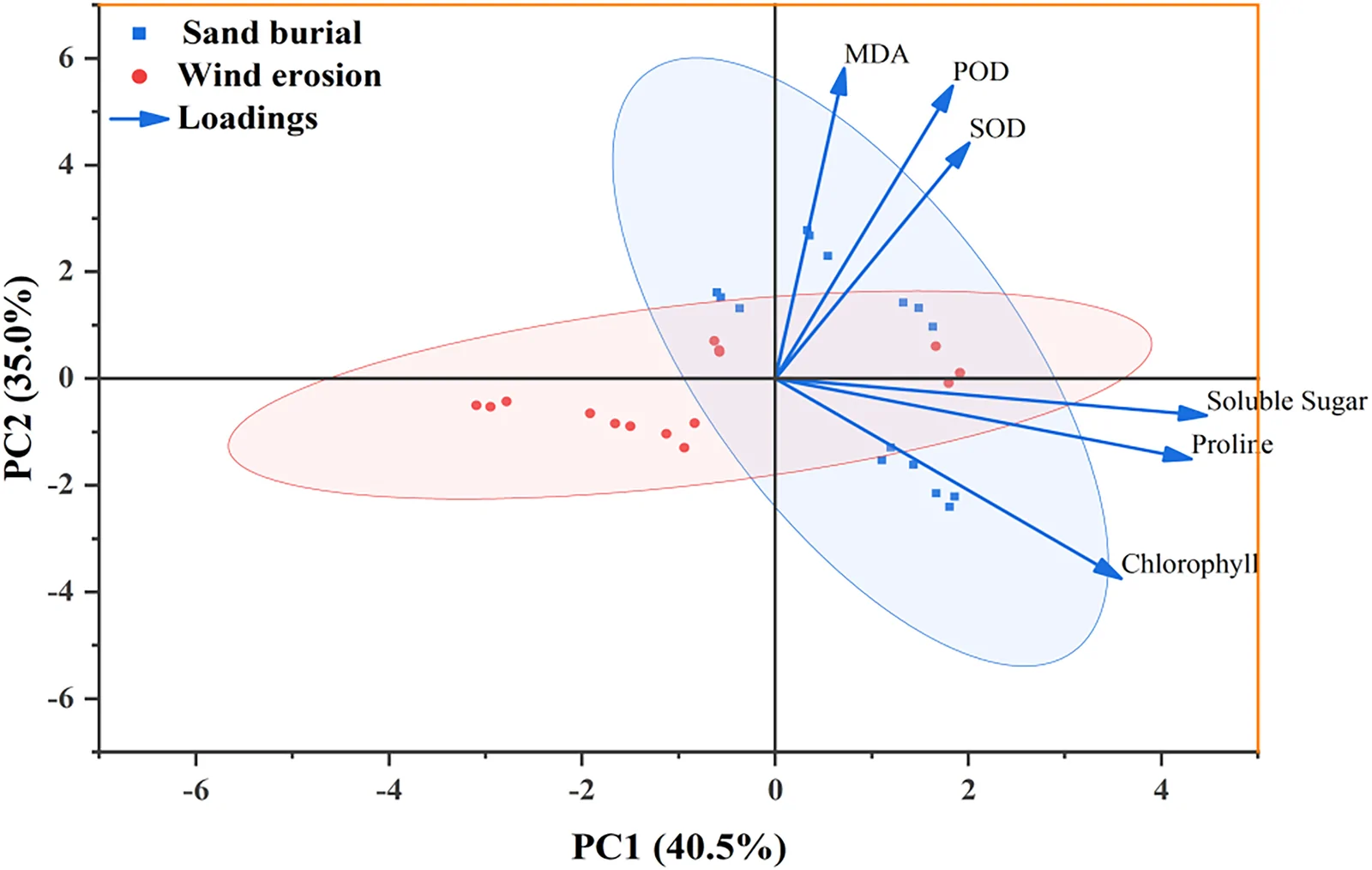
Principal component analysis (PCA) score and loading plot of six physiological indicators of *A. wudanica* under wind erosion (red dots) and sand burial (blue squares) gradients. Blue arrows represent loading vectors; the direction and length of each arrow denote the contribution direction and strength of the corresponding physiological indicator to the principal components. Values in parentheses on the axes indicate the proportion of variance explained by PC1 (40.5%) and PC2 (35.0%).

Separate Spearman correlation analyses showed contrasting patterns of trait coordination under sand burial and wind erosion (**Figure 5**). Under sand burial, chlorophyll, proline, and soluble sugar were significantly and positively correlated with one another, whereas several of these traits were negatively associated with POD or SOD. MDA was strongly positively correlated with POD (ρ = 0.84, *P* < 0.001). Under wind erosion, chlorophyll was strongly positively correlated with proline (ρ = 0.96, *P* < 0.001), and soluble sugar was positively correlated not only with chlorophyll and proline but also with MDA, POD, and SOD. In addition, POD and SOD showed a strong positive correlation (ρ = 0.78, *P* < 0.001). These results demonstrate that the coordination among physiological traits differed markedly between the two disturbance types.

**Figure 5:**
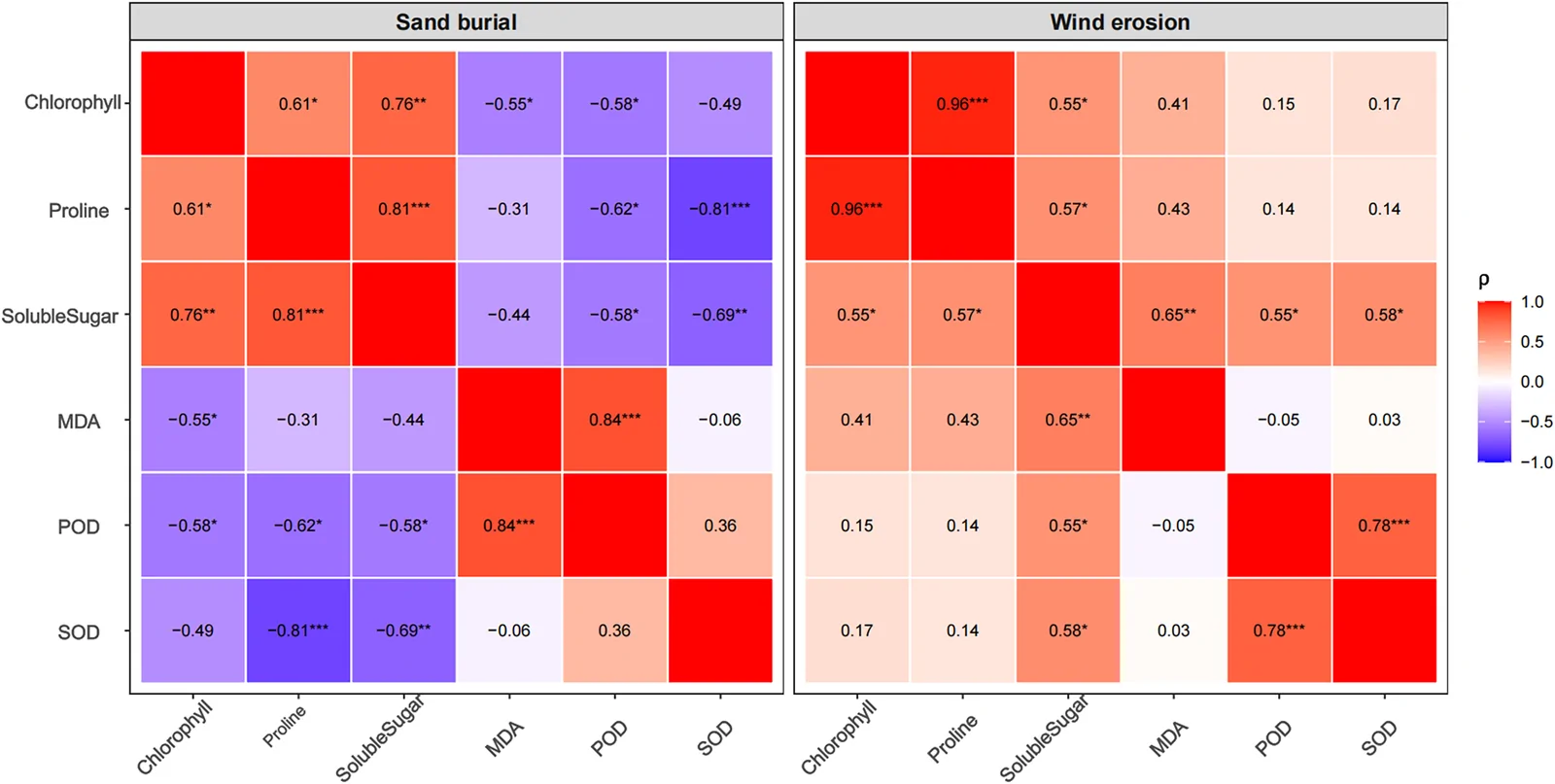
Spearman correlation heatmap of physiological indices of *A. wudanica* under wind erosion and sand burial disturbance. Color intensity represents the correlation coefficient, with red indicating positive correlation and blue indicating negative correlation. Significance levels are denoted by asterisks: *P < 0.05, **P < 0.01, ***P < 0.001.

### 3.3 Disturbance-specific physiological response structures under wind erosion and sand burial

The disturbance-specific correlation patterns, together with PCA and the exploratory path analysis, suggest contrasting physiological response structures under the two disturbance treatments rather than experimentally validated physiological mechanisms (**Figures 6**, **7**). Under sand burial, chlorophyll, proline, and soluble sugar showed close positive covariation, consistent with a response pattern centered on photosynthetic maintenance and osmotic adjustment. Under simulated wind erosion, POD and SOD were strongly associated, while soluble sugar also covaried with MDA, POD, and SOD. Notably, MDA was not significantly correlated with either POD or SOD, indicating that the wind-erosion response was characterized by broader cross-functional associations rather than a discrete and tightly coordinated damage–antioxidant defense module.

**Figure 6:**
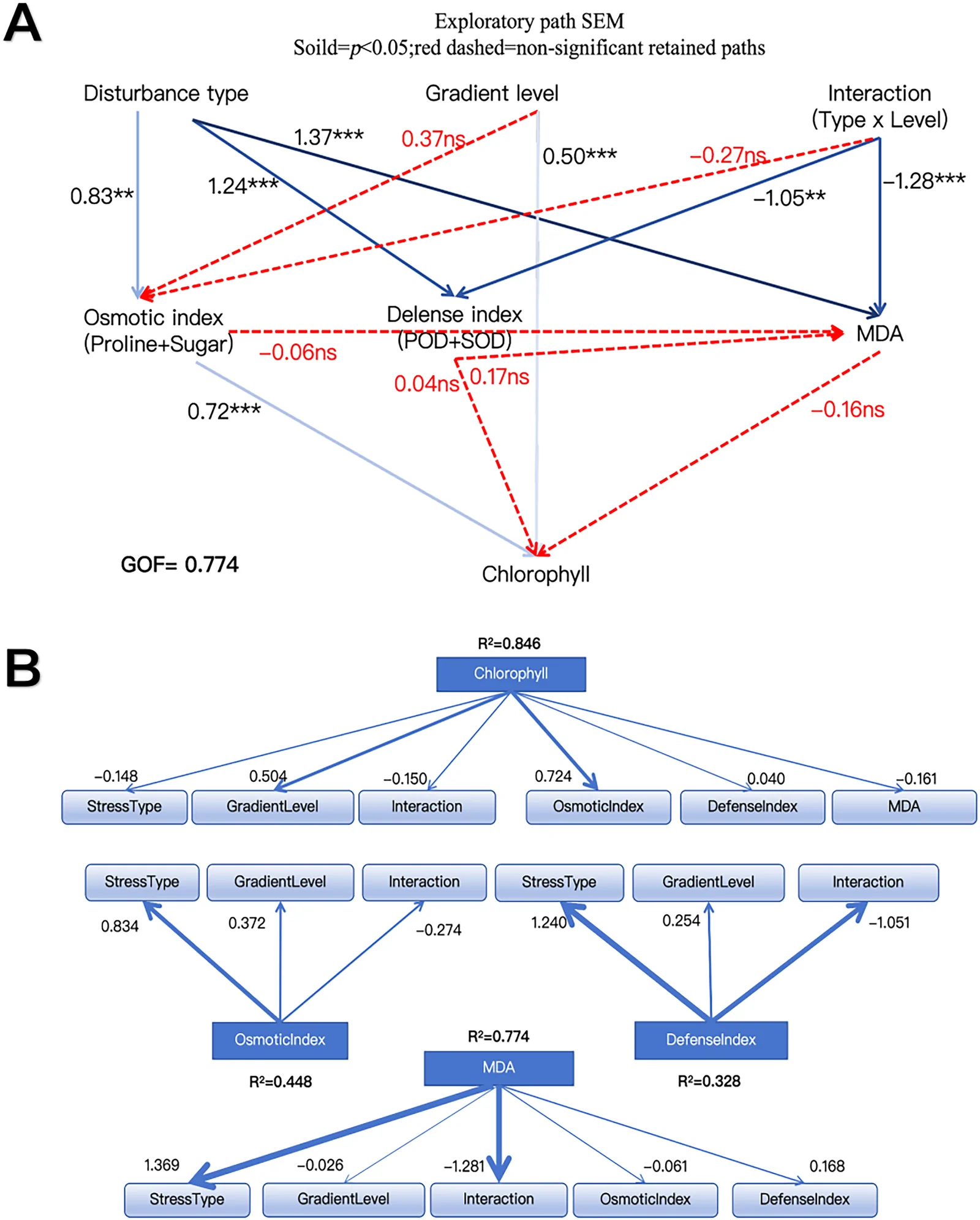
Exploratory path model summarizing standardized associations among disturbance type, disturbance gradient, their interaction, and physiological response dimensions of *A. wudanica*. The model is exploratory and the paths should not be interpreted as experimentally validated causal mechanisms. Path coefficients are standardized. Significance levels are denoted by asterisks: *P < 0.05, **P < 0.01, ***P < 0.001. Panel **(A)** illustrates the overall association structure, and panel **(B)** shows factor-specific contributions.

**Figure 7:**
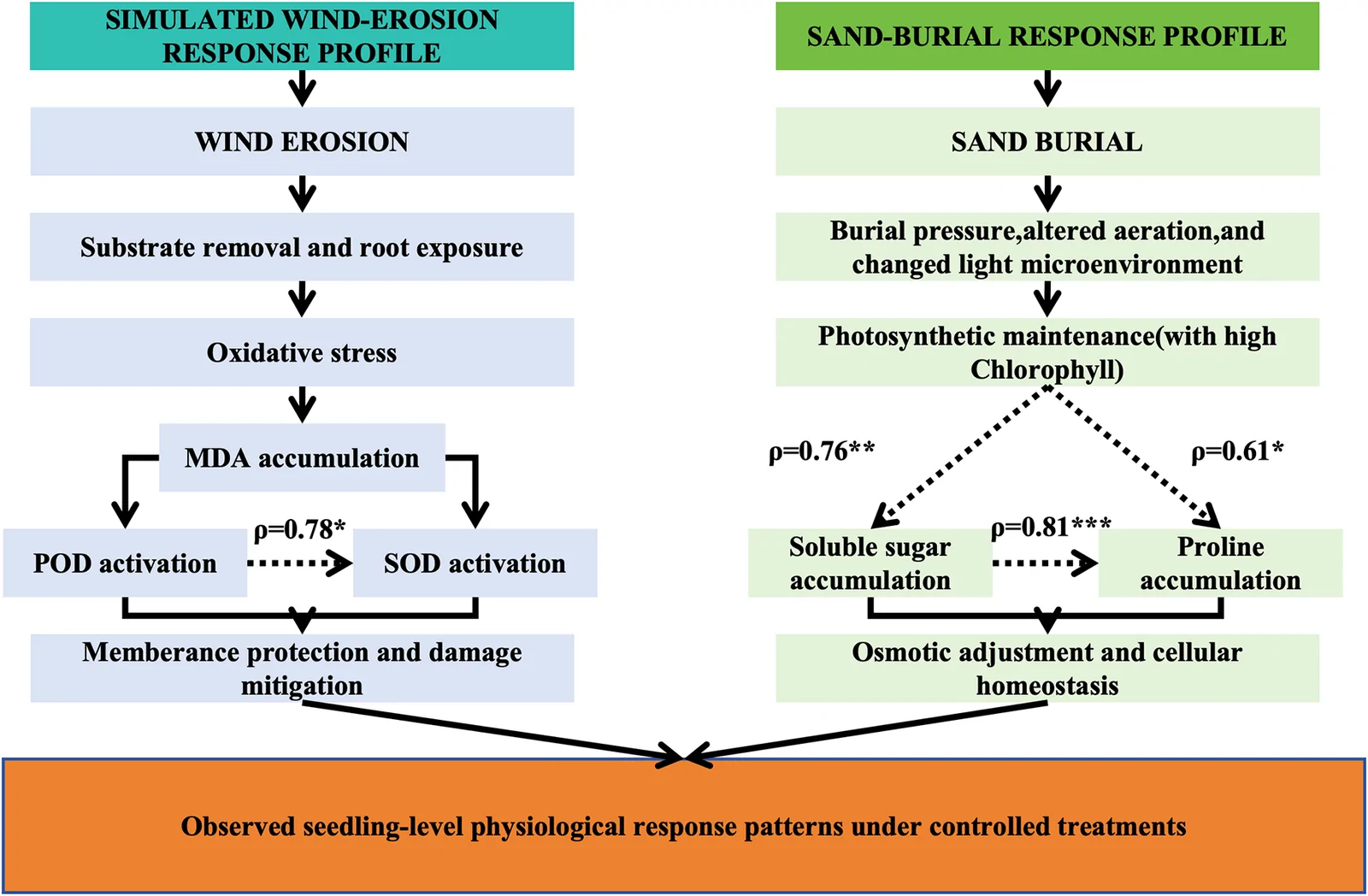
Conceptual summary of treatment-associated physiological response patterns of *Artemisia wudanica* seedlings under simulated wind erosion and sand burial. Solid arrows indicate hypothesized relationships based on the observed treatment responses and previous literature, whereas dashed lines indicate within-treatment Spearman associations. The diagram is intended as a conceptual interpretation of the observed physiological response profiles and does not represent experimentally validated causal, sequential, or switchable regulatory mechanisms. Significance level: *P < 0.05, **P < 0.01, ***P < 0.001.

The exploratory path analysis further indicated associations of disturbance type and the disturbance type × gradient interaction with several physiological response dimensions, including osmotic adjustment, antioxidant defense, and MDA. It also suggested a positive association between osmotic adjustment and chlorophyll and a negative association between MDA and chlorophyll. However, because several fitted paths were not supported by bootstrap confidence intervals and the overall model fit was limited, these relationships are interpreted as exploratory statistical associations rather than direct causal effects or validated physiological mechanisms. Taken together with the univariate and PCA results, the analyses indicate that sand burial and simulated wind erosion were associated with different physiological response configurations: sand burial showed relatively concentrated photosynthetic–osmotic covariation, whereas simulated wind erosion exhibited antioxidant-enzyme responses together with broader cross-functional coupling. These patterns should therefore be regarded as working interpretations of seedling responses under the controlled experimental conditions rather than definitive evidence of physiological mechanisms.

## 4 Discussion

This study characterized physiological responses of *A. wudanica* seedlings to two isolated, field-informed disturbance gradients under controlled pot conditions. Previous studies have documented ecological and morphological responses of this species in sandy habitats, including recruitment and seed-retention traits and differential growth responses to wind erosion and sand burial (Gong et al., 2020; Wu et al., 2020; Liu et al., 2014; Wei et al., 2025). Our contribution is therefore an integrated, gradient-based assessment of six physiological traits using univariate analyses, descriptive PCA, Spearman correlations, and an exploratory path analysis. Across these analyses, simulated substrate removal/root exposure and sand burial were associated with different physiological response profiles. We interpret these differences as treatment-associated seedling response patterns rather than as two validated physiological mechanisms, distinct regulatory logics, or mechanisms demonstrated by the present experiment. Previous studies on psammophytic plants have often focused on the individual effects of wind erosion or sand burial on growth and physiology but have rarely compared their regulatory logic within a unified framework (Qu et al., 2022; Shi et al., 2023; Homberger et al., 2025; Lee et al., 2023). By integrating single-indicator responses with multivariate and coupling analyses, our study not only supports the view that aeolian disturbance exerts complex physiological effects but also suggests that different aeolian disturbance types can induce qualitatively distinct adaptive strategies in sandy-habitat plants.

### 4.1 Contrasting physiological responses to simulated wind erosion and sand burial

Wind erosion and sand burial have often been treated as different stages of the same aeolian disturbance process because they frequently co-occur and alternate in sandy habitats (Qu et al., 2022; Lu et al., 2023; Dinca et al., 2025; Zhu and Li, 2024). Although these processes frequently co-occur or alternate in natural dune systems, they were experimentally isolated in the present study to distinguish their individual physiological effects. Therefore, the contrasts reported here refer to responses to each disturbance applied separately and do not represent physiological responses to combined aeolian disturbance. However, this interpretation may obscure their different physiological consequences. In the present study, significant interaction effects across all six physiological traits, together with the clear separation of treatments in multivariate space, indicate that wind erosion and sand burial impose different functional demands on *A. wudanica*. Therefore, at least for this species, the two disturbance types should be regarded as qualitatively distinct disturbance regimes rather than different points along a single disturbance continuum.

The distinction between these two disturbance regimes can be explained by their different modes of physical disturbance. In the present experiment, the simulated wind-erosion treatment directly imposed substrate removal and the associated exposure of the root system. These changes can disrupt the root–soil interface and may contribute to physiological stress. Under natural wind-erosion conditions, additional factors such as wind force, enhanced desiccation, and mechanical abrasion may further intensify oxidative disturbance and membrane injury, as reported in previous studies (Wang et al., 2022; Gong et al., 2021; Kirschner et al., 2021; Li et al., 2024). This provides a mechanistic explanation for the stronger association of wind erosion with MDA, POD, and SOD in our study. Wind erosion was associated with stronger responses of MDA, POD, and SOD in the univariate and multivariate analyses. However, the disturbance-specific Spearman analysis showed that only POD and SOD were strongly positively correlated, whereas MDA was not significantly correlated with either antioxidant enzyme. This suggests that enzymatic antioxidant defense was tightly coordinated under wind erosion, while lipid peroxidation and antioxidant enzyme activity were partially decoupled across the erosion gradient.

By contrast, sand burial directly added substrate around the seedling base and shallow root zone. Previous studies indicate that burial can modify local light, gas diffusion, mechanical load, and near-surface water and temperature conditions (Shao et al., 2025; Mao et al., 2023). In the present experiment, chlorophyll, proline, and soluble sugar showed significant positive covariation in the disturbance-specific Spearman analysis. This statistical pattern is consistent with a response profile involving photosynthetic and osmotic traits, but it should not be interpreted as direct evidence that these processes were functionally coordinated by a single regulatory pathway. The contrast with simulated wind erosion therefore describes different observed response structures under matched relative gradients rather than a complete mechanistic explanation of the two natural disturbances.

### 4.2 Non-linear and disturbance-specific physiological responses of *A. wudanica*

A second important finding of this study is that the physiological responses of *A. wudanica* did not follow a simple linear disturbance–response pattern. In many studies of abiotic disturbance, increasing disturbance is expected to cause progressive declines in photosynthetic performance and continuous increases in cellular damage. This framework has also been widely applied to aeolian disturbance research (Zhai et al., 2022; Fan et al., 2018; Ma et al., 2014; Qu et al., 2012; Song et al., 2021). However, several indicators in our experiment, particularly chlorophyll and MDA, showed pronounced non-linear fluctuations rather than monotonic changes.

The non-monotonic patterns do not demonstrate that *A. wudanica* restored physiological homeostasis within a specific disturbance range, because measurements were made at the end of the treatment period rather than through time. Similar threshold-dependent responses have been reported in other dune plants. For example, in *Agriophyllum squarrosum*, light sand burial increased survival, plant height, and biomass, whereas deeper burial intensified membrane lipid peroxidation and cellular damage (Li et al., 2015). In coastal dune species, moderate burial can stimulate growth or photosynthetic performance, whereas excessive burial can suppress recovery (Maun, 1998; Perumal and Maun, 2006; Frosini et al., 2012). These studies provide a comparative basis for considering compensatory responses as one possible explanation for the fluctuating patterns observed here, but such compensation was not directly measured in this endpoint experiment.

Studies on psammophytic and disturbance-tolerant plants have shown that moderate disturbance may induce compensatory regulation, including adjustment of photosynthetic pigment content, renewed tissue production, osmotic adjustment, and redistribution of physiological resources (Huang et al., 2022; Liu et al., 2014). In *A. wudanica*, the non-linear responses of chlorophyll and MDA may therefore reflect a dynamic balance between stress injury and compensatory repair. Under low to moderate disturbance, physiological adjustment may partially offset the negative effects of sand movement, whereas under stronger disturbance, membrane damage and metabolic imbalance may exceed the plant’s regulatory capacity. In contrast, persistent declines reported under deeper burial or prolonged erosive disturbance may occur when the disturbance exceeds the compensatory capacity of the plant (Zhang, 1996). Because aeolian disturbance in natural sandy habitats is typically pulsed, spatially heterogeneous, and temporally variable rather than continuously intensifying (Okin, 2025), such non-linear regulation may represent a potentially important seedling-level response to aeolian disturbance, although its ecological relevance under natural mobile-dune conditions remains to be tested.

Within the sand-burial series, Spearman associations among chlorophyll, proline, and soluble sugar define a relatively concentrated photosynthetic–osmotic covariation pattern. Burial can reduce light availability for basal tissues and modify aeration and water conditions around buried organs, and previous studies report chlorophyll maintenance, compatible-solute accumulation, and carbon-allocation changes under burial (Chen et al., 2023; Zhong et al., 2024; Gaile et al., 2021; Ye et al., 2019; Kuroda, 2023; Tian et al., 2025; Renzetti et al., 2025). For instance, *Agriophyllum squarrosum* seedlings increased SOD, POD, and free proline under sand burial (Li et al., 2015), and *Sporobolus virginicus* altered shoot morphology under sediment accretion (Frosini et al., 2012). These studies are consistent with a working interpretation that multiple physiological functions contribute to burial responses, but our correlations alone do not establish a coordinated pathway or resource-reallocation mechanism in *A. wudanica*.

In contrast, wind erosion was more strongly associated with MDA, POD, and SOD, indicating a pathway dominated by oxidative damage signals and antioxidative defense. Root exposure resulting from substrate removal may contribute to physiological stress in the present experiment. In natural wind-eroded environments, associated water loss and mechanical abrasion may further promote reactive oxygen species accumulation and membrane lipid peroxidation, thereby increasing the demand for antioxidant defense (Wang et al., 2022; Huang et al., 2023; Pei and Liu, 2026; Wang et al., 2025; Sood, 2025; Alam et al., 2025; Cannea and Padiglia, 2025). Similar patterns have been observed in other sandy-habitat plants. In *Salix gordejevii*, wind erosion significantly reduced growth, gas exchange, chlorophyll fluorescence, and leaf water status, while altering proline, soluble sugar, SOD, POD, and MDA levels (Ma et al., 2022). Root exposure experiments with Hedysarum scoparium and *Caragana korshinskii* also showed that exposed roots induced excessive oxygen free radicals, increased MDA accumulation, and activated species-specific antioxidant and osmotic regulation pathways (Ma et al., 2023). These results are consistent with our finding that wind erosion in *A. wudanica* was closely linked to membrane lipid peroxidation and antioxidant enzyme activation.

However, *A. wudanica* also differed from some other psammophytes in the way its physiological traits were coordinated. In *Calligonum mongolicum*, moderate sand burial promoted clonal regeneration, whereas wind-eroded rhizomes depended strongly on physiological integration with mother ramets for survival (Fan et al., 2018). In contrast, *A. wudanica* is a local endemic psammophyte inhabiting mobile and semi-mobile dunes, and its adaptation appears to rely more strongly on rapid physiological regulation at the seedling stage. The strong positive correlation between POD and SOD under wind erosion indicates coordinated regulation of antioxidant enzymes. In contrast, the absence of significant correlations between MDA and either POD or SOD suggests that the degree of lipid peroxidation was not tightly synchronized with antioxidant enzyme activity across the erosion gradient. Interestingly, soluble sugar was positively associated with MDA, POD, and SOD, suggesting that osmotic/carbon-related adjustment may participate in the broader physiological response to wind erosion. Unlike species or disturbance conditions in which antioxidant enzymes remain constitutively elevated under long-term disturbance (Kumari et al., 2024), *A. wudanica* appears to activate this system as an inducible emergency module under erosive disturbance. Therefore, its physiological plasticity lies not only in the magnitude of individual trait responses, but also in its capacity to shift physiological response pathways according to disturbance type.

### 4.3 Ecological implications and limitations of disturbance-specific physiological response patterns

Taken together, our results indicate contrasting treatment-associated physiological response profiles in *A. wudanica* seedlings rather than two validated or switchable physiological pathways. Under simulated wind erosion, which directly represented substrate removal and root exposure in this experiment, MDA, POD, and SOD showed comparatively strong responses, while POD and SOD exhibited significant positive covariation. However, MDA was not significantly associated with either antioxidant enzyme after disturbance-specific analysis, indicating that oxidative damage and antioxidant-enzyme responses should not be interpreted as a tightly coordinated damage–defense pathway. Under sand burial, chlorophyll, proline, and soluble sugar showed a more concentrated pattern of positive covariation, consistent with a photosynthetic–osmotic response profile. Previous studies have highlighted the relevance of physiological responses to environmental disturbance in plants (Ji et al., 2023; Zhao et al., 2023). Considered individually, however, these indicators provide only a partial description of the overall physiological response. In the present study, PCA and disturbance-specific correlation analyses, together with the exploratory path analysis as a complementary statistical summary, suggest that the two independently treated seedling groups differed in their multivariate response profiles and patterns of trait covariation. Because these analyses were based on endpoint measurements from separate treatment series, they do not demonstrate causal regulatory pathways, functional coordination, or physiological switching between disturbance types within individual plants. Rather, under the controlled pot conditions used here, simulated substrate removal and sand burial were associated with different, context-dependent physiological response profiles in *A. wudanica* seedlings. Whether these contrasting patterns also occur under natural, sequential, or combined aeolian disturbances requires further experimental validation.

Earlier studies show that *A. wudanica* occurs and persists in dynamic dune habitats through ecological and morphological traits such as recruitment, seed retention, and flexible growth responses (Gong et al., 2020; Wu et al., 2020; Wei et al., 2025). The present physiological measurements may help generate hypotheses about how seedlings respond to contrasting disturbances, but they do not directly explain ecological success, population persistence, or dominance in natural dunes. Likewise, the experiment did not evaluate restoration establishment or management performance, so restoration implications should be treated as possibilities requiring field validation rather than direct recommendations.

The experiment also did not expose the same individuals to wind erosion and sand burial simultaneously or sequentially. Consequently, it cannot determine whether the effects of the two disturbances are additive, synergistic, antagonistic, or dependent on disturbance order and recovery history. The contrasting response profiles observed under the two isolated treatments therefore should not be described as switchable strategies within individual plants. Future factorial and event-sequence experiments will be required to test whether response patterns change when erosion and burial occur together or in alternating sequences. In addition, the relatively small number of independent biological replicates per treatment (n = 3) limits statistical power and the precision of treatment-effect estimates; therefore, the physiological response patterns should be interpreted cautiously.

An important limitation of the present experiment is that the imposed wind-erosion and sand-burial treatments did not fully reproduce the temporal dynamics of natural aeolian disturbances. Under field conditions, wind erosion and sand burial typically occur as episodic events associated with periods of strong wind, followed by intervals of relatively weak disturbance, recovery, or subsequent disturbance. In contrast, our experiment maintained prescribed erosion or burial depths by inspecting treatment levels daily and adjusting the sand surface only when deviations from the target depth occurred. This design allowed the effects of disturbance magnitude to be evaluated under standardized conditions, but it necessarily simplified the temporal variability of natural aeolian processes.

## 5 Conclusion

This study demonstrates that *A. wudanica* seedlings exhibit disturbance-specific physiological response and trait-covariation patterns under controlled wind-erosion and sand-burial treatments. Sand burial was characterized by positive covariation among chlorophyll, proline, and soluble sugar, consistent with a photosynthetic–osmotic response pattern. Wind erosion showed broader cross-functional coordination, including strong POD–SOD coupling and positive associations of soluble sugar with oxidative-damage and antioxidant traits. These findings indicate that wind erosion and sand burial reorganize physiological trait relationships in different ways rather than eliciting a single generalized stress-response pathway. However, because the experiment used young seedlings under controlled, maintained, and isolated disturbance treatments, these patterns should not be directly extrapolated to mature plants or natural dune populations. Future studies incorporating field conditions, multiple developmental stages, and simultaneous or sequential erosion–burial events are needed to determine the ecological generality of these physiological responses.

## Data Availability

The original contributions presented in the study are included in the article/**Supplementary Material**. Further inquiries can be directed to the corresponding author.
