# Supplementary material for: Physiological response strategies of pioneer endemic species Artemisia wudanica along an aeolian disturbance gradient in semi-arid active sand dunes
Source: Front Plant Sci. 2026 Sep 30;17:1981049. doi: 10.3389/fpls.2026.1981049 (PMC13661353; doi:10.3389/fpls.2026.1981049)
Supplement: Supplementary file 1 [file Table1.docx]

**Supplementary Information**

**Table 1. The degree of wind erosion and sand burial treatment, as well as daily treatment**

|  | 1 | 2 | 3 | 4 | 5 |
| --- | --- | --- | --- | --- | --- |
| Sand burial | 0 | 1.7 | 3.3 | 5 | 6.7 |
| Daily treatment | 0 | 0.06 | 0.11 | 0.17 | 0.22 |
| Wind erosion | 0 | 2 | 4 | 6 | 8 |
| Daily treatment | 0 | 0.07 | 0.13 | 0.2 | 0.27 |

****Table 2. Analytical methods used for physiological measurements****

| ****Indicator**** | ****Method**** | ****Reference/standard**** | ****Instrument**** | ****Unit**** | ****Enzyme activity definition**** |
| --- | --- | --- | --- | --- | --- |
| Total chlorophyll, including chlorophyll a and b | Double-wavelength colorimetric method | NY/T 3082-2017 | Mettler ML204 analytical balance; PERSEE T6 UV–visible spectrophotometer | mg·g^-1^ | Not applicable |
| Proline | Ninhydrin colorimetric method | Wang, Principles and Techniques of Plant Physiological and Biochemical Experiments | Mettler ML204 analytical balance; Thermo Fisher Scientific Multiskan GO 1510 microplate reader | mg·kg^-1^ | Not applicable |
| Soluble sugar | Sulfuric acid–anthrone colorimetric method | Wang, Principles and Techniques of Plant Physiological and Biochemical Experiments | Mettler ML204 analytical balance; Thermo Fisher Scientific Multiskan GO 1510 microplate reader | mg·g^-1^ | Not applicable |
| MDA | Thiobarbituric acid colorimetric method | Wang, Principles and Techniques of Plant Physiological and Biochemical Experiments | Mettler ML204 analytical balance; PERSEE T6 UV–visible spectrophotometer | μg·g^-1^ | Not applicable |
| POD | Guaiacol colorimetric method | Wang, Principles and Techniques of Plant Physiological and Biochemical Experiments | Mettler ML204 analytical balance; PERSEE T6 UV–visible spectrophotometer | U·g^-1^·min^-1^ | One unit was defined as a change of 0.01 in A470 per minute per gram tissue in each milliliter of reaction system |
| SOD | Nitroblue tetrazolium photoreduction method | Wang, Principles and Techniques of Plant Physiological and Biochemical Experiments | Mettler ML204 analytical balance; PERSEE T6 UV–visible spectrophotometer | U·g^-1^ | One unit was defined as the amount of enzyme required to inhibit NBT photoreduction by 50% |

**Table 3. Summary of Two‑way ANOVA**

|  | **Indicator** | **disturbance Type_p** | **Gradient Level_p** | **Interaction_p** |  |
| --- | --- | --- | --- | --- | --- |
| 1 | Chlorophyll | 1.705848e-02 | 4.219626e-12 | 5.778483e-06 |  |
| 2 | Proline | 2.946822e-18 | 6.754881e-14 | 5.667444e-15 |  |
| 3 | Soluble Sugar | 2.329292e-15 | 2.946451e-16 | 1.362545e-15 |  |
| 4 | MDA | 1.827247e-08 | 1.398293e-12 | 1.440629e-11 |  |
| 5 | POD | 9.434797e-10 | 4.206206e-18 | 2.116209e-18 |  |
| 6 | SOD | 6.345620e-14 | 2.412133e-10 | 3.735693e-17 |  |

**Table 4. Simple-effect analyses and Tukey HSD comparisons for the disturbance type × relative disturbance level interaction**

| **Trait** | **Disturbance type** | **df1** | **df2** | **F** | **Exact P** | **Partial η^2^** |
| --- | --- | --- | --- | --- | --- | --- |
| Chlorophyll | wind erosion | 4 | 20 | 43.410 | 1.38×10^-9^ | 0.897 |
| Chlorophyll | sand burial | 4 | 20 | 54.044 | 1.93×10^-10^ | 0.915 |
| Proline | wind erosion | 4 | 20 | 114.948 | 1.68×10^-13^ | 0.958 |
| Proline | sand burial | 4 | 20 | 174.952 | 2.94×10^-15^ | 0.972 |
| Soluble sugar | wind erosion | 4 | 20 | 306.386 | 1.24×10^-17^ | 0.984 |
| Soluble sugar | sand burial | 4 | 20 | 104.612 | 4.11×10^-13^ | 0.954 |
| MDA | wind erosion | 4 | 20 | 18.024 | 2.06×10^-6^ | 0.783 |
| MDA | sand burial | 4 | 20 | 145.573 | 1.74×10^-14^ | 0.967 |
| POD | wind erosion | 4 | 20 | 252.789 | 8.14×10^-17^ | 0.981 |
| POD | sand burial | 4 | 20 | 455.616 | 2.47×10^-19^ | 0.989 |
| SOD | wind erosion | 4 | 20 | 198.228 | 8.74×10^-16^ | 0.975 |
| SOD | sand burial | 4 | 20 | 128.203 | 5.90×10^-14^ | 0.962 |
